# Combining the clinical frailty scale with grip strength for the identification of frailty among end-stage kidney disease patients

**DOI:** 10.1093/ckj/sfae232

**Published:** 2024-07-26

**Authors:** Kyra Lamberink, Paul A Rootjes, Yolande M Vermeeren, Arthur D Moes, Tizza P Zomer

**Affiliations:** Dialysis Centre Apeldoorn, Gelre Hospitals, Apeldoorn, The Netherlands; Dialysis Centre Apeldoorn, Gelre Hospitals, Apeldoorn, The Netherlands; Dialysis Centre Apeldoorn, Gelre Hospitals, Apeldoorn, The Netherlands; Dialysis Centre Apeldoorn, Gelre Hospitals, Apeldoorn, The Netherlands; Dialysis Centre Apeldoorn, Gelre Hospitals, Apeldoorn, The Netherlands

To the Editor,

Frailty and sarcopenia are both associated with a worse prognosis and higher risk of mortality [1]. As frailty is prevalent among end-stage kidney disease (ESKD) patients, these patients may benefit from advance care planning (ACP) [2]. A reliable, straightforward tool to screen for frailty in this population is lacking, which may compromise the identification of frail patients [3]. To assess frailty, the clinical frailty scale (CFS) is an easy to use assessment tool, however previous research indicated that this tool in its current form is not reliable enough to use in daily clinical practice [2]. The assessment of grip strength (GS), on the other hand, can be used to identify individuals at risk for sarcopenia. The aim of the current study was to investigate the combined performance of the CFS and GS as a screening tool to identify frailty in ESKD patients.

In this cross-sectional study, haemodialysis, peritoneal dialysis, pre-dialysis patients and patients receiving conservative therapy from Dialysis Centre Apeldoorn, The Netherlands, were included. Methods have been described in detail elsewhere [2]. The Frailty Index (FI) by Searle *et al*. [4] was used as the golden standard and patients with a score ≥0.25 were considered frail. The GS was extracted from the FI for independent assessment alongside the CFS. The CFS scores were assigned by nephrologists who were not specifically trained in its use [5]. A non-frail outcome for the CFS and GS was scored as non-frail. Conversely, a frail outcome for either the CFS or GS constituted a frail score. Sensitivity, specificity, positive predictive value, negative predictive value and area under the curve (AUC) were calculated to evaluate the combined diagnostic performance of the CFS and GS.

A total of 144 patients were included. Mean age was 67.4 ± 13.5 years and 56 (38.9%) were females. Overall, 72 (50.0%) patients were treated by haemodialysis, 13 (9.0%) by peritoneal dialysis, 6 (4.2%) received conservative treatment and 53 (36.8%) were pre-dialysis patients. According to the FI, 60 (41.7%) patients were considered frail. Removing the GS from the FI had no effect on the performance of the FI. The combined GS and CFS ≥4 demonstrated a sensitivity of 93.3%, while the GS and CFS ≥5 combination yielded a sensitivity of 90.0% (Table 1). Specificity values were 67.9% and 71.4%, respectively, and the AUC was identical for both CFS cut-off points (0.81).

**Table 1:** Test performances of the CFS and GS assessment compared to the FI.

	CFS ≥4	CFS ≥5	GS	CFS ≥4 + GS	CFS ≥5 + GS
Sensitivity, % (95% CI)	63.3 (49.9–75.4)	50.0 (36.8–63.2)	80.0 (67.7–89.2)	93.3 (83.8–98.2)	90.0 (79.5–96.2)
Specificity, % (95% CI)	81.0 (70.9–88.7)	91.7 (83.6–96.6)	73.8 (63.1–82.8)	67.9 (56.8–77.6)	71.4 (60.5–80.8)
PPV, % (95% CI)	70.4 (59.5–79.4)	81.1 (66.9–90.1)	68.6 (59.9–76.2)	67.5 (60.2–74.1)	69.2 (61.4–76.2)
NPV, % (95% CI)	75.6 (68.5–80.6)	72.0 (66.4–76.9)	83.8 (75.4–89.7)	93.4 (84.5–97.4)	90.9 (82.2–95.6)
AUC (95% CI)	0.72 (0.63–0.81)	0.71 (0.62–0.80)	0.77 (0.69–0.85)	0.81 (0.73–0.88)	0.81 (0.73–0.88)

PPV, positive predictive value; NPV, negative predictive value.

This study investigated the CFS combined with GS as a screening tool for the identification of frailty in ESKD patients. The combined CFS and GS showed an improved sensitivity and AUC and decreased specificity compared with the CFS alone [2]. These results may have been affected by the lack of specific training in the use of the CFS by the nephrologists. Despite the increased sensitivity of the combined CFS and GS, the specificity decreased. This may result in unnecessary ACP, which is both financially and time consuming. Therefore, the CFS combined with GS in its current form is not yet suitable for use as a frailty screening tool in daily clinical practice and further research is warranted. Nevertheless, the CFS in combination with GS may be used as a first step to identify frailty, after which patients are subsequently subjected to a comprehensive geriatric assessment. Furthermore, specific training of nephrologists in handling the CFS might improve test performance.

In conclusion, combining the CFS with GS to identify frailty results in improved test performance compared with the CFS alone. However, the decrease in specificity indicates that more research is warranted before the CFS can be used as a single frailty screening tool in daily clinical practice.

## Data Availability

The data for this study are available from the corresponding author after reasonable request.
